# User Experience of Extended Reality Treatment for Visuospatial Neglect Among Patients and Informal Caregivers: Qualitative Interview Study

**DOI:** 10.2196/80136

**Published:** 2026-04-20

**Authors:** Eileen Bousché, Hanne Huygelier, Tanja CW Nijboer, Knowledge Broker Neglect Study Group, Eugenie Brinkhof

**Affiliations:** 1 Experimental Psychology, Helmholtz Institute, Utrecht University, Heidelberglaan 1, Utrecht, 3584 CS, The Netherlands, +31 30 253 4700; 2 Department of Brain and Cognition, KU Leuven, Leuven, Belgium; 3 See Acknowledgments

**Keywords:** visuospatial neglect, visual scanning training, extended reality, virtual reality, augmented reality, qualitative data

## Abstract

**Background:**

Visuospatial neglect (VSN) is a cognitive disorder following a stroke, where individuals fail to perceive or respond to stimuli on the contralesional side of space. Visual scanning training (VST) is the recommended treatment in clinical guidelines.

**Objective:**

This qualitative study explored how patients (N=10) and informal caregivers (N=8) perceived the usability and potential implementation of 3 extended reality (XR)–based serious games to enhance VST—1 in virtual reality and 2 in augmented reality—as tools for VST. These technologies not only enhance patient engagement but also enable detailed data collection to monitor therapeutic progress.

**Methods:**

The themes and feedback were compared with themes and feedback from VSN therapists from a previous study: (1) suitability for VSN rehabilitation, (2) applicability, (3) motivation, (4) guidance, (5) versatility, and (6) detailed insights in game performance.

**Results:**

Highlights were that patients reported high engagement and enjoyment, with many expressing willingness to use the games in both clinical and home settings. Informal caregivers supported these findings and emphasized the motivational value of the games. Additionally, both groups noted the importance of accessible instructions and technical support.

**Conclusions:**

Although XR technology offers potential in neurorehabilitation, a uniform solution may not suit all users. This study showed the importance of including diverse end-user groups in development for usability, acceptance, and implementation. Successful integration of XR in rehabilitation requires customizable features, structured support, and attention to the differing roles of caregivers. Further research is needed to evaluate the clinical effectiveness and optimize patient-tailored applications of XR in VSN treatment.

## Introduction

One of the fundamental cognitive skills required for daily activities, such as locating personal belongings, navigating traffic, and engaging in social interactions, is the ability to scan the environment. Unfortunately, for individuals who have experienced a stroke, this ability is often impaired due to visuospatial neglect (VSN). VSN is a common poststroke cognitive disorder characterized by a lateralized attentional deficit [1-4]. This deficit manifests in behaviors such as bumping into objects, overlooking food on one side of the plate, or grooming only one side of the body. The consequences of VSN negatively affect functional independence, activities of daily living, and societal participation [5-8]. Informal caregivers often bear a significant burden, compensating for the functional deficits caused by VSN [9]. Clinical guidelines recommend visual scanning training (VST) as a primary intervention [10]. VST is a compensatory therapy designed to train systematic eye and head movements toward the contralesional side [11-13]. The therapy dose is quite intensive, as it requires time commitment up to 40 hours over 5 weeks [11]. Moreover, patients’ lack of awareness of their VSN, a condition known as anosognosia [14-16], may diminish their motivation and engagement in therapy. This is particularly problematic when the effectiveness or quality of the learned compensatory strategy is difficult to assess visually using current forms of VST. If a device could be used that not only provides individualized, interactive training but also objectively indicates how effectively the strategy is being applied, this could significantly enhance both monitoring and therapeutic outcomes.

Advancements in extended reality (XR) technology, especially virtual reality (VR) and augmented reality (AR), offer promising new approaches to VSN rehabilitation [17-19]. VR immerses individuals in fully simulated environments via specialized headsets, whereas AR integrates digital elements into real-world settings, allowing for interaction within familiar surroundings. When combined with serious games [20], a gamified form of therapy, XR not only enhances patient engagement, enjoyment, and usability [21-23] but also enables detailed data collection to monitor therapeutic progress [24,25]. Studies further support the potential of XR for both assessing and improving visuospatial attention after stroke. Immersive and semi-immersive VR environments have been shown to elicit and measure neglect-like behavior [26-28]. Moreover, a VR-based exploration task has demonstrated positive effects on spatial attention and engagement [29], and AR tasks have been used to strengthen compensatory scanning behavior in functional contexts [30,31]. In parallel, growing evidence shows the importance of adaptability and personalization in neglect rehabilitation. For instance, an adaptive cueing protocol, in which visual cues were gradually reduced as patients improved, resulted in meaningful gains in VSN-related functional outcomes [32]. Although this paradigm was developed outside XR, it provides a strong conceptual foundation for implementing performance-dependent, adaptive task adjustments within XR-based VST.

Despite these encouraging developments, current XR interventions for VSN often do not proceed to the implementation process. A crucial step required to eventually move from the development lab toward clinical practice is to involve end users. Thorough evaluation of end-user perspectives, especially those of patients and caregivers, is required to secure the implementation of VR and AR therapy [33,34].

Building on previous therapist evaluations [35], this study incorporated patient and informal caregiver feedback into the iterative development of 3 serious games for VSN rehabilitation (1 VR-based and 2 AR-based). In this study, we collected perspectives of patients and informal caregivers and assessed to what extent they were distinct from the perspectives of therapists on the same 3 games reported by Bousché et al [35]. We aimed to assess whether these 2 end-user groups also mentioned the same 6 key findings from therapist evaluations: (1) suitability for VSN rehabilitation, (2) applicability, (3) motivation, (4) guidance, (5) versatility (ie, patient’s needs), and (6) detailed insights in game performance. Variance in responses among groups will emphasize the need for inclusion of different end-user groups in the development process.

## Methods

### Participants

The first group of end users consisted of patients with VSN admitted to De Hoogstraat Rehabilitation Center for inpatient rehabilitation between April and October 2021. Inclusion criteria were as follows: (1) admitted to the neurology ward, (2) presence of VSN diagnosed by a neuropsychologist or screened by a therapist using the Balloons Test [36] or Catherine Bergego Scale (CBS) [37], and (3) age 18 years or older. The second group of end users consisted of informal caregivers of participating patients. Inclusion criteria were as follows: (1) age 18 years or older and (2) a familial or social relationship with the patient. Epilepsy was the sole exclusion criterion for all participants because of potential triggers for seizures in the serious games.

### XR Serious Games

Three XR serious games were administered. First, the AR Virtual Museum used a Microsoft HoloLens 1 (1268×720 pixel per eye; 60 Hz; 35° FOV). This game promotes visual search by projecting virtual paintings onto real-world walls. Participants moved around the room to locate paintings, which transformed into short video clips upon discovery [30]. Second, VR HEMIRehApp employed an Oculus Rift CV1 (1080×1200 pixel per eye; 90 Hz; 110° FOV) with handheld controllers. Designed to retrain spatial attention toward the contralesional side, the game places participants in a virtual farm setting with tasks such as harvesting crops and feeding animals. It was initially developed for right-hemispheric stroke patients with intact language abilities [38, 39], and its validity and usability were confirmed in a pilot study [24]. Third, the Balloon Popping game, developed by Holomoves, used a HoloLens 2 (2048×1080 pixel per eye; 60 Hz; 52° FOV). This AR-based intervention integrates visual search training with physical movement. Virtual balloons appear around the player, who must locate and “pop” them using finger gestures. Auditory (verbal instructions) and visual (green directional arrows) cues aid the task. Participants played 3 mini games: simple balloon popping, image pairing, and word formation.  

### Outcome Measures: Interviews

A semistructured interview (Textbox 1) was designed by the authors, which consisted of 15 qualitative and 2 quantitative questions where the participant could rate an aspect of the XR game. The design was informed by the System Usability Scale [40], a widely used instrument for rapid quantitative assessment of usability. To obtain a deeper understanding of participants’ experiences, we supplemented the scale with additional targeted questions addressing VST, user experience (eg, own performance, equipment use, cybersickness), usability (eg, playing the game autonomously, during therapy), and preference (eg, liking, improvement) of the games. The interview was recorded for analysis. Informal caregivers received a similar interview, without item 16 (practicing visual search outside therapy). Additionally, the items on practicing autonomously were rephrased for helping the patient at home or at the rehabilitation center.

Textbox 1. **Interview on user experience, usability, and preference questionnaire. Participants rated difficulty and enjoyment on a scale from 0 to 10, where higher scores indicate greater difficulty or enjoyment.**

**User experience**

1. What did you think of the game?

2a. What went well?

2b. What went less well (game-related or personal performance)?

3. How did the headset feel on your head?

4. Was there anything about the headset or controllers that you found uncomfortable?

5. How did you experience moving around in the virtual environment?

6. Some people feel warm or need time to adjust. How was this for you? How did it feel to play the game?

**Usability**

7a. Did you find the game difficult or easy (rate on a scale from 0=very easy to 10=very difficult)?

7b. What influenced this?

8. What could make playing the game easier or more difficult for you?

9. What did you think about the duration of the game?

10a. Can you imagine using this game on your own for extra practice?

10b. Why or why not?

11. If we were to offer a 2-week training program to practice daily with this game, would you be willing to participate?

12a. Would you recommend this game to fellow rehabilitation patients?

12b. Why or why not?

**Preference**

13. How enjoyable did you find the game (rate on a scale from 0=not enjoyable at all to 10=very enjoyable)?

14. What aspects made the game particularly enjoyable to play?

15. What do you think could make the game even more enjoyable?

16. Do you currently practice searching and scanning for objects outside of therapy?

17. Is there anything that would prevent you from wanting to play this game?

### Procedure

Patients were first informed about the study by researchers and invited to participate. Interested patients were scheduled to play their first game within 2 weeks. Before gameplay, the researcher reviewed the information letter and study procedure with the patient. For patients with aphasia, a speech therapist facilitated communication using simplified phrasing, repetition techniques, questioning, and pictorial techniques. The AR games were played either while walking, sitting, or rolling in a wheelchair or office chair. The VR game was played while participants were seated. After the patient completed the games, their informal caregiver was invited to participate and play the games as well. Each participant played all games once during one-on-one sessions with the researcher within a period of 3 weeks in a quiet room. Researchers assisted with headset placement, provided instructions, and answered questions. Throughout gameplay, the researcher closely monitored participants for signs of discomfort, fatigue, or cybersickness, such as dizziness, nausea, or disorientation. Participants were explicitly instructed to report any discomfort at any time, and gameplay was paused or stopped if adverse effects were observed or reported. Game order was counterbalanced, with each session lasting 10 to 15 minutes, including instructions. Each game was played once. After each game, a 15-minute semistructured interview was conducted with each participant.

### Patient Data

The following patient data were collected from electronic patient files: age, sex, time poststroke onset (in days), stroke type (ischemic, hemorrhagic), and lesion side (left, right, bilateral). Functional scores were also collected for strength in the upper and lower extremities using the Motricity Index (MI) [41], global cognitive function using the Montreal Cognitive Assessment [42], level of communication using the Dutch Aphasia Foundation (Stichting Afasie Nederland) [43], and independence in activities of daily living using the Barthel Index [44]. Two neuropsychological tests were used to assess VSN severity: the Balloons Test [36,45] and the CBS [37].

### Analyses 

To explore the user experience, applicability, and suggestions of the patients and informal caregivers, the ratings were described and the qualitative data were analyzed. Audio files of the interviews were transcribed using Microsoft Word and later adjusted by the researchers to make a coherent story. Then, a deductive coding scheme was formed based on 6 key findings from therapists’ evaluations: (1) suitability for VSN rehabilitation, (2) applicability, (3) motivation, (4) guidance, (5) versatility (ie, patient’s needs), and (6) detailed insights in game performance [35], following the approach described by Boeije et al [46]. These themes served as a preliminary analytic framework, based on the assumption that variation in perspectives across stakeholder groups would show the importance of incorporating multiple end-user voices in XR development. For the present study, each of the 6 themes was further differentiated into “positive,” “negative,” and “neutral” subcategories to capture the valence of participants’ remarks and “suggestions” to facilitate a more nuanced comparison across user groups (Multimedia Appendix 1). All interviews were coded by 1 researcher, with 2 other researchers taking samples from both groups (ie, patients and informal caregivers). The samples were then further discussed in the research team to reach consensus on the coding process. After reaching consensus, all interviews were coded again based on the agreed-upon criteria. Qualitative findings were descriptively triangulated with test observations and available clinical data (eg, neglect severity, lesion side) to support interpretation and identify converging or diverging patterns, without formal quantitative hypothesis testing.

### Ethical Considerations

This study was approved by the Medical Ethics Committee of the University Medical Center Utrecht and the Ethical Committee of De Hoogstraat Rehabilitation Center (reference number: 21/706). All procedures were conducted in accordance with the Declaration of Helsinki. All participants provided written informed consent prior to participation. The data consisted of anonymized patient and interview data, which were collected and processed in a manner that ensured participant privacy and confidentiality. Participants did not receive financial or other compensation.

## Results

### Patient Data

A total of 12 patients with VSN were recruited and provided informed consent to participate in the study. However, 2 patients withdrew before participation due to fatigue, reduced cognitive capacity, lack of motivation, and difficulty recalling study details or scheduled sessions. Of the 10 remaining patients (age 49‐77 y; 70% male), 3 played all games, 6 played 2 games, and 1 played 1 game. Additionally, 10 informal caregivers were recruited (age 45‐75 y; 25% male), of whom 8 participated in this study (n=5 played all games; n=2 played 2 games; n=1 played 1 game). Interview excerpts were translated from Dutch to English to serve as examples in this publication.

Table 1 presents individual patient demographic and clinical characteristics. The majority (n=7) had experienced ischemic stroke, 2 had experienced a hemorrhagic stroke, and 1 patient had a cerebral abscess. Lesion lateralization was right hemispheric in 6 patients, left hemispheric in 3, and bilateral in 1 patient. Most (n=8) were in the subacute phase of recovery; the rest were in the acute phase (n=2). The patient group presented with VSN that was mild or not detectable using conventional paper-and-pencil tests. However, clinical observations as scored with the CBS revealed the full range of VSN severity. CBS data for 7 patients were available, indicating mild VSN for 10% (CBS<10), moderate VSN for 30% (CBS<20), and severe VSN for 30% (CBS>20). For those patients without CBS scores, 1 patient exhibited moderate neglect symptoms on the Balloons Test (Subtest B score<17; lateralization balance score<40%), and for 2 other patients, clinical observations indicated the presence of VSN. Although VSN was underrepresented on standardized assessments, the group nonetheless included patients across the entire spectrum of VSN. Cognitive screening indicated that all patients had cognitive impairment, ranging from impaired to severely impaired levels, with all scores falling below the clinical cutoff (Montreal Cognitive Assessment score<26). Motor function, using the MI, showed that patients on average had moderate motor impairment (0‐33 severe; 34‐66 moderate; and 67‐99 mild/normal). In terms of functional independence, patients were generally highly dependent on others for daily care. Based on their scores on the Barthel Index, the group as a whole fell within the range corresponding to “severely dependent” (5-9), although individual scores ranged from “completely dependent” (0‐4) to “moderately dependent” (10-14). This reflects a patient population with substantial functional needs and limited autonomy in daily living.

**Table 1.** Patient demographic and clinical characteristics at admission to the rehabilitation center.^a^

ID	Age (y)	Sex	Time post onset (d)	Hemisphere	Etiology	MI^b^ Arm (0‐99)	MI Leg (0‐99)	MoCA^c^ (0‐30)	SAN^d^ (0‐7)	BI^e^ (0‐20)	CBS^f^ (0‐30)	Balloons (A and B=L0-10/R0-10; LB^g^ 0%‐50%)
1	53	Male	25	Left	Ischemic	18	59	17/30	5	8/20	17.5	—^h^
2	60	Male	22	Left	Hemorrhagic	0	0	Partial (aphasia) visuospatial 2/5; orientation 3/6	2	2/20	—	A=L10/R10; B=L10/R10; LB=50%
3	49	Male	13	Right	Hemorrhagic	56	75	18/30	7	4/20	—	A=L10/R10; B=L5/R8; LB=38.5%
4	64	Male	72	Right	Ischemic	29	75	23/30	7	8/20	22.5	A=L4/R10; B=L0/R3; LB=0%
5	56	Male	9	Right	Ischemic	0	83	25/30	7	12/20	13.3	A=L10/R10; B=L7/R10; LB=41.2%
6	68	Male	4	Right	Ischemic	78	78	21/30	5	13/20	27	A=L9/R10; B=L4/R8; LB=34%
7	77	Female	7	Right	Recurrent ischemic	76	64	23/30	5‐6	18/30	16.25	A=L10/R10; B=L8/R7; LB=46.7%
8	52	Female	9	Left	Ischemic	0	—	Not assessed (aphasia)	2	7/20	9	—
9	61	Male	14	Right	Cerebral abscess	83	91	24/30	7	13/20	—	A=L10/R10; B=L10/R10; LB=50%
10	74	Female	14	Bilateral	Ischemic	76	83	4/30	3‐4	12/20	24.4	—

a Values are presented as raw scores unless otherwise specified. Aphasia was assessed using the SAN test. Complete SAN data were available for all participants.

b MI: Motricity Index.

c MoCA: Montreal Cognitive Assessment.

d SAN: Stichting Afasie Nederland (aphasia).

e BI: Barthel Index.

f CBS: Catherine Bergego Scale.

g LB: lateralization balance.

h Missing data.

### Suitability for VSN Rehabilitation

**Figure 1.**
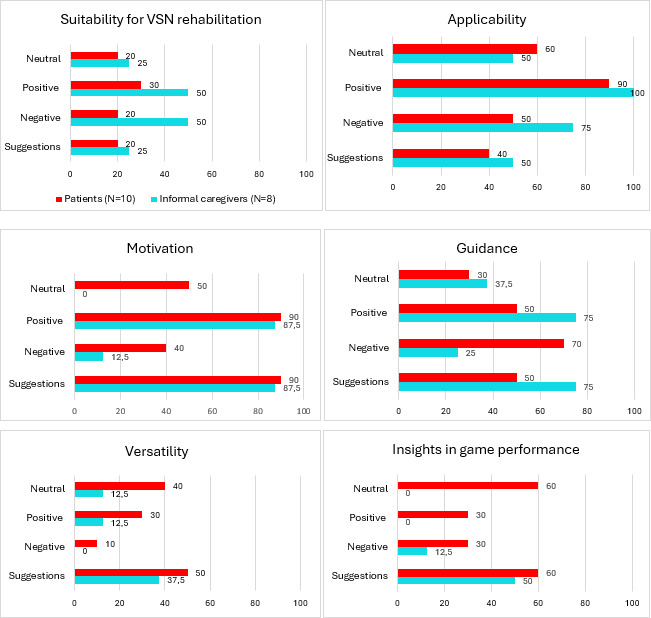
The coding scheme reflected in percentages of patients (N=10) and their informal caregivers (N=8) who gave neutral, positive, and negative responses and suggestions. VSN: visuospatial neglect.

The participants’ perceptions of the games’ suitability for VSN rehabilitation were varied (Figure 1). Patients provided fewer comments on this aspect compared with other categories and even fewer than their informal caregivers. Among informal caregivers, the distribution of positive and negative responses was equal. An informal caregiver mentioned that for their loved one, training would be appropriate, while it was too easy for a healthy person such as themselves. It was also suggested to improve clarity regarding the game’s main objective and ensure that patients understand the added value of playing. There were also suggestions for improving the suitability of the games. For example, one informal caregiver mentioned that adjustments need to be made for suitability for neglect, because currently, the targets were only right in front of the player.

…I thought: now something is going to happen, but most of the targets were actually right in front of me. Those are things to which more attention is paid to: who will be playing next? Okay, this patient has left-sided neglect, then we need to place more targets on the left. Sometimes also behind the patient, so they really have to turn their head and search for where it is.

[Informal caregiver03]

### Applicability

The applicability of VR and AR games to the target patient population was an important topic for both patients and their informal caregivers. Ninety percent (9/10) of patients and 100% (8/8) of informal caregivers made positive remarks about the feasibility and applicability of these games (Figure 1). Patients reported that the games were easy to play and that the duration of gameplay was sufficient. Patients also indicated feeling comfortable while playing and were able to move freely while seated in an office chair or wheelchair.

Similarly, most informal caregivers found the games intuitive and of appropriate duration when played themselves; however, some expressed concerns regarding the potential difficulty of gameplay duration for patients with VSN, thus making a distinction between their own experiences and the anticipated challenges faced by patients.    

Despite overall positive responses, 50% (5/10) of patients also expressed concerns regarding the applicability of the games. One patient wondered whether older individuals would be able to engage with the games effectively.

*I really like the idea. But I can imagine that for older people it might be quite difficult to use.*

[Patient05]

Even more informal caregivers were concerned (6/8, 75%), with issues related to game content (eg, difficulty with multitasking), safety (eg, potential distraction leading to reduced awareness of surroundings), and patient-specific factors (eg, individuals with memory impairments might struggle with certain games).

*…You get lots of information to read, so you have a lot to process. This could be hard for people who have had a stroke….*

[Informal caregiver08] 

*No I wasn’t distracted by the paintings, but the patient might bump into something.*

[Informal caregiver07]

*…But with the patient it will not be that easy. I think when making a turn, the patient might fall.*

[Informal caregiver03]

*In my case, my memory is good. But for people with a stroke, memory is often affected, right? If you make the game more difficult, I think it might become too demanding for them.*

[Informal caregiver05]

Furthermore, it was observed that 1 patient encountered difficulties while engaging with AR Balloons and VR HEMIRehApp, due to the specific motor demands of these games (eg, pointing and using a controller). Conversely, AR Museum proved to be more suitable for this patient, as it only required eye fixations. Although the patient was not able to finish the games, the patient still had positive views on this type of technology.

Additionally, 40% (4/10) of the patients provided suggestions for improving applicability. Patients questioned whether it was feasible to integrate the games within the busy schedule of the rehabilitation center, proposing that a single session might be appropriate, without being specific about an exact therapy dose.

*…But it has to be feasible alongside other therapy sessions. Well…it doesn’t need to be a problem, but when you have many [therapy sessions] it could become difficult. But when you would receive it [the equipment] for a single day, it would be okay.* 

[Patient08]

Among informal caregivers, 50% (4/8) suggested modifications to enhance applicability, primarily focusing on safety (eg, using a large, empty space to minimize the risk of collisions) and interface adjustments (eg, a more salient cursor to keep track of one’s actions in the game).

*…I noticed quickly that you really have to poke through the balloon, but I wonder how patients will handle this, that you really need to poke through the balloon.…Maybe the game could make the cursor or pointer more salient so it’s clearer what they are actually interacting with. For me it wasn’t a problem, but it might be for others.*

[Informal caregiver03]

### Motivation

Both groups were asked about the perceived likability of the games and their motivation to continue playing or reengage with the games. Although participants expressed a range of motivation-related sentiments (neutral, positive, and negative), the content of these responses showed little variation. Overall, patients (9/10, 90%) and informal caregivers (7/8, 87.5%) reported similarly positive perceptions of the games’ motivational aspects. The most frequently mentioned motivational factor was positive affect, as participants indicated that they enjoyed playing the games. For instance, 1 patient noted that, beyond the therapeutic necessity of the games, engaging with them provided a welcome distraction from routine therapy.

*…as for the game element, I think many people would enjoy it simply because it’s something different and engaging.*

[Patient05]

Patients appreciated the physical engagement involved, such as making head movements, searching, and scanning, which the games facilitated.

*I really appreciated the movements involved: searching, moving the head, and scanning. I saw many positive aspects in it.*

[Patient 09]

*Moving around was easy. You knew when the music stopped the game was finished, so you had to keep going until you found another target.*

[Patient01]

Informal caregivers mentioned the appeal of the games’ colors and dynamic elements, as well as their informative nature. 

*In the last game, I especially liked that the deer was moving that made it more engaging. Overall, I thought the game was well designed.*

[Informal caregiver10]

*I really enjoyed it, you receive information and things are explained, for example about a painting. I didn’t necessarily recognize anything personally, but I still liked that something was being told and that you could learn from it.*

[Informal caregiver08]   

Negative feedback was more prevalent among patients (4/10, 40%) than among informal caregivers (1/8, 12.5%). Informal caregivers primarily mentioned the difficulty level of the games. Patients, however, reported concerns such as inadequate gamification of therapeutic elements within the game design and difficulties in locating objects. 

*At this moment I would not yet recommend it, because it is still in the research phase. First, people should practice with everyday situations, things they normally encounter and tend to neglect in daily life. That should be mastered first.*

[Patient03]

*…Yes, because I couldn’t find them easily. If the targets were easier to locate, your attention would be drawn to them more, and you would probably be able to continue for longer.*

[Patient04]

*…I think it has to do with the fact that you just have to click away two things each time. Not much else happens, you know?*

[Informal caregiver07]   

Suggestions for enhancing motivation were largely consistent across groups, with 90% (9/10) of patients and 87% (7/8) of informal caregivers proposing modifications such as incorporating diverse themes (eg, zoo, art), increasing difficulty levels (eg, more targets, time constraints), and introducing additional objectives (eg, puzzles, object-chasing tasks).

*Maybe there could be multiple themes, or a slightly more difficult level. For example, combining two themes in one task. That would add more challenge, because you would need to look more actively for different types of objects.*

[Patient09]

*Maybe there could be a time limit, so you know in advance that you have two or three minutes. But it has to be realistic, it should still be achievable for someone with a condition like mine.*

[Patient05]

### Guidance

The theme regarding guidance encompassed tutorials or support from another person, from the game itself, or from documents. Positive remarks from both patients and informal caregivers were that the instructions within the games were clear. The graphics and targets were visible, and the instructional voice was audible.  

*The instructions were explained clearly while I was seated, and everything was said out loud and shown on screen, so I didn’t even have to read them. I could just start playing right away.*

[Patient01]  

According to the patients (7/10, 70%), negative aspects of guidance were the instructions for gameplay, controllers, or the act of selecting targets, as well as the lack of feedback on their gaming performance.

*Maybe the next time, once you know what the intention is, it will be easier.*

[Patient10]

*What you have to remember is that with the green leaf you need to use the lower button instead of the upper one and with the orange leaf it’s the other way around.*

[Patient05]

Negative aspects according to informal caregivers (2/8, 25%) were, for example, that the in-game instructions were not sufficiently noticeable or understandable for patients.

*I think that for people who have difficulties understanding due to a brain injury, a bit more guidance at the beginning would be helpful, clearly explaining what to do. After that, they would probably be able to manage.*

[Informal caregiver8]

Suggestions from patients (5/10, 50%) included allowing participants to practice more to accurately remember the instructions. Another interesting suggestion was to enhance cues and feedback related to game performance.

*I don’t know if it’s technically possible, but if you could see what I’m seeing, it might be helpful if you could say something like* “*your gaze is in the right place” or* “*look up” or* “*look down,” especially since our eyes and pupils are apparently being measured.*

[Patient06]

Additionally, 75% (6/8) of the informal caregivers had suggestions for improving the guidance. A recommendation was to give the instructions a more therapeutic focus, for example, by suggesting the use of a different hand to select targets.

*…You really need to indicate “use your other hand.” It’s not that you will automatically use your left. If you have left-sided neglect, use your left hand. Maybe there could be text in between, indicating which finger or hand to use, to stimulate that.*

[Informal caregiver09]

### Versatility (ie, Patient’s Needs)

* *Next to suitability, versatility or tailoring the games to individual patients’ needs was less often mentioned, compared with other themes from the coding scheme. Versatility of the serious games was perceived positively by 30% (3/10) of patients and 12% (1/8) of informal caregivers. One negative aspect was mentioned by a single patient.

*For me, the music could be different. I can imagine that younger people might really like this, but we are from a different generation, so I would prefer another type of music.*

[Patient06]

Suggestions regarding versatility were made by half of the patients (5/10, 50%) and just over a third of the informal caregivers (3/8, 38%). Patients and informal caregivers primarily suggested adapting game themes to personal preferences, such as allowing users to choose their own virtual environment.

*Maybe there could be multiple themes, or a slightly higher level of difficulty, for example by combining two themes in one task. That would add more challenge, because you would have to search for different types of objects.*

[Patient09]

*There should be more variation so people can choose what they want to do, for example, different environments or more animals appearing in the game.*

[Informal caregiver05]

*For example, my brother loves farm life. If the game included a video about a farm and the animals, he would probably find it much more interesting.*

[Informal caregiver01]

### Detailed Insights in Game Performance 

A difference was found between the 2 groups in mentioning the insights derived from gameplay. Whereas patients expressed a clear desire to know how they performed in the games, informal caregivers showed less interest, with only 13% (1/8) mentioning it as a negative aspect. Suggestions for game performance insights primarily involved displaying accuracy or total score. A notable suggestion was to include an option that allows users to compare their current performance with previous results or the performance of other players.

*Just showing the number of points or the percentage of correct answers is enough for me. Something like a graph would also work.*

[Patient 03]

*…What matters is whether you did it correctly or not.*

[Patient 02]

***Researcher:** Would you like to know how well you did in the game?*

***Patient:** Yes, of course.*

***Researcher:** Would you also like to see how others performed, or just compare your own results next time?*

***Patient:** Yes actually, I’d like to see everyone’s scores. That would be fun.*

***Researcher:** Then you could gradually try to catch up with the top score, like “Jan,” who did better.*

***Patient:** Yes, I like that.*

[Patient10]

### Other Findings 

Other findings revealed that informal caregivers evaluated the games from both their own perspective and that of the patients. Informal caregivers often elaborated on imagining how playing the game would be for the patient.

*Yes, I think it went well. I had no problems, but I can imagine that a patient, like the patient with left-sided neglect, would find it harder to distinguish, for instance, where a balloon is located.…I can imagine that a patient might have more difficulty and would need to think constantly.*

[Informal caregiver03]

*I can imagine that if someone isn’t really into games, like my brother, who doesn’t like them at all, they might quickly lose interest.*

[Informal caregiver01]

This dual perspective is comparable to the approach taken by therapists who primarily considered the patient’s viewpoint. Interestingly, therapists leaned more toward patient-centered observations, while informal caregivers balanced their own experience with their perception of the patient’s experience. 

To increase the total time for dedicated training, it was explored whether patients would use the serious games at home or at the rehabilitation center, preferably with the help of an informal caregiver. Patients expressed willingness to practice at home if provided with the necessary equipment. Similarly, they were open to using the headsets at rehabilitation centers if readily available. However, reasons for not doing so included having other matters to attend to at home or preferring to spend time with friends and family at the rehabilitation center. Informal caregivers (6/8, 75%) expressed a willingness to support home practice, provided it fit within their daily schedules. However, their willingness to assist in rehabilitation centers was more divided (4/8, 50%), often dependent on practical considerations such as work commitments or the belief that therapy should primarily be managed by health care professionals.

*It depends on the location. Not here at the Hoogstraat Rehabilitation, that would take too much time. But if he had one at home, I would be willing to do it. If I had to come here every day with him, then I think the care staff would need to do this more often.*

[Informal caregiver01]

Lastly, no adverse effects were reported. The hardware and software were generally well received (7/10, 70% patients; 6/8, 75% informal caregivers). However, a few participants (2/10, 20% patients; 2/8, 25% informal caregivers) reported initial difficulty adjusting to the VR headset.  

*It took a moment to get used to it, a bit of dizziness, and then I returned and,“phew.”*

[Patient02]

Realistic VR scenarios, such as fish catching over a virtual lake, induced vertigo for one informal caregiver who reported fear of heights after the session. 

*I personally have a bit of a fear of heights, which may sound strange, but in that water scene it really felt as if I was above the water. When I looked down, I thought, “wow.”*

[Informal caregiver07]

## Discussion

This study assessed the user experience of patients with VSN and their informal caregivers and compared the findings to the themes frequently mentioned by VSN therapists in a previous study by Bousché et al [35]. The participants engaged in 3 serious games, 1 VR and 2 AR games, based on VST for VSN.

### Principal Findings

Participants’ perceptions regarding the suitability of the games for VSN rehabilitation were varied. Patients generally made fewer comments on this aspect compared with informal caregivers, who showed an equal distribution of positive and negative feedback. Some informal caregivers noted that the training would be suitable for their loved ones but too easy for healthy individuals, and others suggested improving the clarity of game objectives and tailoring the games specifically for neglect (eg, adjusting target placement). In terms of applicability, both groups found the games feasible and user-friendly, although some expressed concerns regarding the duration and potential difficulties for patients with VSN. Suggestions for improvement included better customization for older individuals and ensuring safe use in clinical settings. It seemed to be easier for both groups to talk about applicability and feasibility than the more therapeutic properties of the games, something that is obviously not a problem for VSN therapists.

Next to the therapeutic aspects, motivational aspects were also evaluated. Incorporating gamification elements into XR rehabilitation can increase patient motivation and engagement [31,47-49]. Gamification VST was positively perceived by both patients and informal caregivers. The most frequently mentioned motivation indicator was positive affect, as participants enjoyed the gameplay and appreciated the physical engagement. However, some patients expressed concerns about insufficient gamification of therapeutic elements and difficulty locating objects. Informal caregivers suggested increasing the difficulty level to sustain interest. This is consistent with therapists’ views, which were positive regarding motivational aspects but questioned whether players would pick up the game again. Suggestions for enhancing motivation were similar across groups (patients and informal caregivers), including incorporating diverse themes (eg, zoo, art), increasing difficulty levels, and introducing additional objectives (eg, puzzles).

Both groups appreciated the clear in-game instructions and the visibility of graphics that accompanied the instructions. However, more patients than informal caregivers reported challenges related to guidance, such as difficulty with controller usage and the lack of performance feedback. Morse et al [50] pointed out the importance of integrating clear guidance within XR interventions, as well as including feedback mechanisms to support user engagement. Therapists took it a step further; almost half of the therapists suggested improving the in-game tutorials, instructions for therapists, and protocols for handling the equipment.

Additionally, tailoring the games to the patient’s individual needs was mentioned less frequently compared with other themes, suggesting that further adaptation may enhance the therapeutic experience. VR interventions can be effective when the level of immersion is adapted to patient needs [28]. Tailoring by both patients and informal caregivers was targeted towards suggestions on adjusting themes to the liking of the patient, in that they could choose their own virtual environment, while therapists leaned more toward adjusting the games to different patient types and clinical requirements (eg, patient’s mobility or sensitivity to sensory input).

Interestingly, we found differences between patients and informal caregivers regarding game performance insights. Patients expressed a desire to receive feedback on their gameplay, while informal caregivers showed limited interest, with one mentioning not being interested. Suggestions to improve game performance insights included displaying accuracy or total scores and incorporating a feature that allows users to compare their current performance with previous results or with others. The interest and suggestions were limited compared with the evaluations of therapists. They mentioned accuracy, temporal measures, strategy, motor measures, or combinations thereof [35].   

This pattern is noteworthy in light of the fact that patients with VSN frequently present with anosognosia or reduced awareness of their deficits [14-16]. One might therefore expect limited interest in performance feedback if patients believed they were performing adequately. The opposite pattern observed in this study suggests that interactive XR-based tasks may facilitate a degree of online awareness of performance during task execution, even when more generalized or offline insight remains impaired [14]. The immediate and goal-directed nature of XR gameplay may make errors or omissions more salient to patients than conventional assessment tasks. Although this interpretation remains speculative, it aligns with theoretical accounts distinguishing between online performance monitoring and global awareness of deficits, and it points to the potential of XR environments to support self-monitoring processes without necessarily increasing cognitive burden.

Our study revealed that informal caregivers evaluated the games from both their own perspective and that of the patients. This dual perspective is different from the approach taken by therapists [35], where therapists primarily considered the patient’s viewpoint. Interestingly, therapists leaned more toward patient-centered observations, while informal caregivers balanced their own experience with their perception of the patient’s experience. This difference emphasizes the distinct roles and perspectives of informal caregivers compared with health care professionals. The importance of involving informal caregivers in rehabilitation is further supported by a randomized controlled trial, which found that caregiver participation in VR training improved patient outcomes in unilateral neglect [51]. They therefore recommended trained informal caregivers to provide VR guidance and supervision to patients who have unilateral neglect.

We explored whether patients could independently use the serious games at home, aiming to intensify VST by increasing the therapy dose as described by Pizzamiglio et al [11]. According to Morse et al [50], end users were positive and interested in this type of telerehabilitation for VSN. In the current study, patients expressed willingness to practice at home if provided with the necessary equipment. Similarly, they were open to using the headsets at rehabilitation centers if readily available. Since informal caregiver participation can improve therapeutic outcomes [51,52], they were asked whether they could aid the patients to play at home. Informal caregivers generally expressed a willingness to support home practice, provided it fit within their daily schedules. However, their willingness to assist in rehabilitation centers was more divided, often dependent on practical considerations such as work commitments or the belief that therapy should primarily be managed by health care professionals.

In a systematic review by Lundin et al [53], the importance of monitoring and reporting adverse effects in VR interventions was advocated. In our study, no adverse effects were reported by both patients and informal caregivers. The headsets and VR controllers were generally well received, although some participants initially struggled with headset adaptation. One informal caregiver with a fear of heights experienced mild vertigo during a realistic scenario, calling attention to the need for monitoring cybersickness in XR interventions, specifically in VR.

To conclude, consistent themes across user groups were identified. These findings demonstrate the value of involving diverse end-user groups when designing XR serious games for health care, particularly for VSN therapy. Including patients, their informal caregivers, and therapists throughout the development process [54] can enhance usability and therapeutic relevance, as supported by studies on VR-based rehabilitation [51,52,55].

### Strengths and Limitations

* *A strength of this study is that it involved patients and their informal caregivers in the development process of different types of XR technology for VSN therapy. The aim was not only to eventually optimize the technology and tailor the games to the patient’s needs but also to investigate the potential of including informal caregivers in the therapy and letting the patient work autonomously, as well as make the feedback comparable to the previous study with therapists. The diversity of end users ensures that all relevant groups were involved in the development and evaluation process. Another strength of this study is the inclusion of a representative patient sample, capturing the full range of relevant clinical impairments. The sample included patients with cognitive impairment ranging from mild to severe, VSN severity spanning from very mild to severe as measured by the CBS, and moderate motor impairments as assessed by the MI. This broad clinical spectrum enhances the ecological validity and generalizability of the findings to broader rehabilitation populations. Additionally, the sample size was somewhat larger and with a broader age range than comparable studies [30,31,50]. 

A potential limitation of this study concerns the assessment of VSN for participant inclusion. Conventional standardized measures were not systematically incorporated, which makes it possible that some participants were already relatively well trained or that neglect symptoms were no longer apparent during static, paper-and-pencil tests typically used in clinical practice. Consequently, variability in neglect severity across participants may have influenced their interaction with the XR games. While no clear patterns emerged, there were indications that participants with more pronounced neglect or right-hemispheric lesions required more guidance during gameplay, which may have shaped their user experience. In addition, the presence of aphasia in some participants may have affected the depth and clarity of their verbal feedback, despite facilitation by a speech therapist. Although care was taken to support communication, responses from participants with aphasia may have differed in richness or nuance compared to those without language impairments, potentially influencing the qualitative findings. Another potential limitation is the monocenter design of the study. The rehabilitation center involved is progressive in its use of science and technology, which may have positively influenced participants’ attitudes toward XR-based interventions. Furthermore, no questionnaire was administered to assess prior experience with digital technology, which limits insight into possible familiarity effects and introduces the risk of selection or contextual bias related to the study setting. Additionally, gameplay sessions were relatively short, which likely limited the occurrence of attentional fluctuations, fatigue, or more pronounced manifestations of VSN during use. As a result, the findings should be interpreted as a snapshot of user experience at a single point in time. Future research should therefore investigate patient and caregiver experiences across longer or repeated gameplay sessions to better assess sustained engagement, usability, and the potential therapeutic value of XR-based interventions over time.

### Clinical Implications 

This study contributed to the first steps toward implementing XR technology in rehabilitation centers. Our findings suggest that integrating XR-based therapeutic interventions into rehabilitation programs for VSN can significantly improve patient engagement and therapy adherence. Given the positive user experience reported by both patients and informal caregivers, the use of VR and AR games appears promising as a supplementary tool for VST. However, to enhance clinical applicability, it is crucial to address challenges related to guidance, customization, and motivation. Specifically, rehabilitation centers should consider integrating these technologies in a way that accommodates both patients and their informal caregivers, providing clear instructions and adaptable game settings. Furthermore, recognizing the varying levels of willingness among informal caregivers to support therapy sessions in different settings is essential for successful implementation.

### Conclusion

This study reported the need for a nuanced understanding of how various end users perceive and interact with XR-based therapeutic interventions. It showed the importance of involving different end users for the development process to become more inclusive and tailored, ultimately enhancing the usability and therapeutic potential of XR applications for VSN. Future research should continue to explore the efficacy and patient-tailored approaches to optimize XR interventions in neurorehabilitation.

## Supplementary material

10.2196/80136 Multimedia Appendix 1 Coding scheme.

## Abbreviations

AR: augmented reality
CBS: Catherine Bergego Scale
MI: Motricity Index
VR: virtual reality
VSN: visuospatial neglect
VST: visual scanning training
XR: extended reality
