# Supplementary material for: User Experience of Extended Reality Treatment for Visuospatial Neglect Among Patients and Informal Caregivers: Qualitative Interview Study
Source: JMIR Rehabil Assist Technol. 2026 Apr 20;13:e80136. doi: 10.2196/80136 (PMC13094804; doi:10.2196/80136)
Supplement: Multimedia Appendix 1 [file rehab-v13-e80136-s001.docx]

Appendix 1. Coding Scheme

**General Coding Rules**

- Assign the most concrete code(s) possible to each response.
- Multiple codes may be assigned to a single response if applicable.

## Coding scheme

- **777** = residual category / does not fit within the coding scheme
- **888** = not interpretable
  - e.g., “could be more user-friendly” or “it is not sensitive enough” → not interpretable
- **999** = no answer to the question / information not relevant

### 1 Suitability for neglect rehabilitation

**Definition:** At first glance, the game appears to achieve its intended purpose.

Use code **1** if the patient makes a general remark about the face validity of the game that cannot be classified under one of the subcategories below. Otherwise, code using one of the following categories:

- **1.1** Positive
- **1.2** Negative
- **1.3** Suggestions

### 2 Applicability of the Games

**Definition:** *Group level.* The game can be applied across different therapeutic approaches, patient populations, diagnostic purposes, and settings.

Use code **2** if the patient makes a general remark about the applicability of the game that cannot be classified under one of the subcategories below.

**2.1 Positive**

- **2.1.1** User experience
- **2.1.2** Technological experience (novice users)
- **2.1.3** Hardware
- **2.1.4** Cybersickness (side effects)

**2.2 Negative**

- **2.2.1** User experience
- **2.2.2** Technological experience (novice users)
- **2.2.3** Hardware
- **2.2.4** Cybersickness (side effects)

**2.3 Suggestions**

- **2.3.1** User experience
- **2.3.2** Technological experience (novice users)
- **2.3.3** Hardware
- **2.3.4** Cybersickness (side effects)

### 3 Motivation Induced by the Games

**Definition:** The extent to which the game motivates the player to start playing, continue playing, and the player’s affective response to the game.

Use code **3** if the patient makes a general remark about motivation elicited by the games. Otherwise, use the categories below.

**3.1 Positive**

- **3.1.1** Affect
- **3.1.2** Willingness to start playing
- **3.1.3** Willingness to continue playing

**3.2 Negative**

- **3.2.1** Affect
- **3.2.2** Willingness to start playing
- **3.2.3** Willingness to continue playing

**3.3 Suggestions**

### 4 Guidance During Gameplay

**Definition:** Support provided during gameplay, including tutorials, instructions, guidance, and manuals.

Use code **4** if the patient makes a general remark about guidance (e.g., instructions, tutorials) that cannot be classified under one of the subcategories below. Otherwise, code using one of the following categories:

**4.1 Positive**

- **4.1.1** Assistance from another person
- **4.1.2** Software-based assistance
- **4.1.3** Manual

**4.2 Negative**

- **4.2.1** Assistance from another person
- **4.2.2** Software-based assistance
- **4.2.3** Manual

**4.3 Suggestions**

### 5 Variety Within the Games

**Definition:** *Individual level.* Adaptations in software or hardware, themes, patient needs, and customization.

Use code **5** if the patient makes a general remark about variety in the game (e.g., adaptations, themes, patient needs) that cannot be classified under one of the subcategories below.

**5.1 Positive**

- **5.1.1** Technological experience (novice users)
- **5.1.2** Hardware
- **5.1.3** Software

**5.2 Negative**

- **5.2.1** Technological experience (novice users)
- **5.2.2** Hardware
- **5.2.3** Software

**5.3 Suggestions**

- **5.3.1** Technological experience (novice users)
- **5.3.2** Hardware
- **5.3.3** Software

### 6 Insights Into Game Performance

**Definition:** Monitoring of game performance within or through the game, including feedback and rewards.

Use code **6** if the patient makes a general remark about insights into game performance provided by the game. Otherwise, use the categories below:

- **6.1** Positive
- **6.2** Negative
- **6.3** Suggestions
